# The *GABPB1* gene A/G polymorphism in Polish rowers

**DOI:** 10.2478/v10078-012-0012-x

**Published:** 2012-04-03

**Authors:** A. Maciejewska-Karłowska, A. Leońska-Duniec, P. Cięszczyk, M. Sawczuk, J. Eider, K. Ficek, S. Sawczyn

**Affiliations:** 1 Department of Genetics, University of Szczecin, Poland.; 2 Department of Physical Culture and Health Promotion, University of Szczecin, Poland.; 3 Galen Medical Center, Bieruń, Poland.; 4 Academy of Physical Culture and Sport, Gdańsk, Poland.

**Keywords:** GABPB1, GABP, NRF2, gene polymorphism, rowing

## Abstract

Nuclear respiratory factor 2 (NRF2), also referred to as the GA-binding protein (GABP) transcription factor, is a key transcriptional activator of many nuclear genes which encode a wide range of mitochondrial enzymes. The variants of the GABPB1 gene encoding the beta1 subunit of NRF2 protein have been associated with physical performance, particularly endurance. The aim of this study was to confirm the possible importance of the A/G polymorphism (rs7181866) in intron 3 of the GABPB1 gene in Polish rowers. The study was carried out on 55 Polish rowers and sedentary individuals, to evaluate the possible relationships between genotype and physical performance. DNA was extracted from buccal cells donated by the subjects. Genotyping was carried out by PCR-RFLP. The results revealed that the frequency of the GABPB1 A/G genotype (89.09% AA; 10.91% AG, 0% GG; vs. 97.69% AA; 2.31% AG; 0.00% GG) %; P = 0.012) and G allele (5.50% vs. 1.17%; P = 0.014) was significantly higher in the rowers compared to controls. The results suggest that the GABPB1 gene can be taken into consideration as a genetic marker in endurance athletes. However, these conclusions should be supported with more experimental studies on other GABPB1 polymorphisms and other genes in elite endurance athletes.

## Introduction

Mitochondria are a part of cells that convert pyruvate into energy for muscular contractions. An organism’s capacity for energy production is usually adapted to the energy requirement. Regular endurance training causes a rapid increase of human muscle capacity for oxidative energy production, as indicated by measurements of the density of mitochondria in certain cells (Turner et al., 1997; Tonkonogi et al., 2000; Baar et al., 2002; Niklas et al., 2010). However, the process by which systematic endurance exercise stimulates biogenesis of mitochondria has not yet been completely understood (Baar et al., 2002; Eynon et al., 2009).

Mitochondria have their own genome, which encodes only 13 respiratory subunits of the almost 100 proteins that constitute the enzyme complex of the mitochondrial respiratory chain (Clayton, 1991). As a result, nuclear genes must provide the majority of products required for mitochondrial oxidative functions and mitochondrial biogenesis. Additionally, they encode factors that control mitochondrial DNA transcription, translation, and replication (Baar et al., 2002; Gleyzer et al., 2005). In a series of previous studies, Scarpulla and his colleagues (Evans and Scarpulla, 1990; Virbasius and Scarpulla, 1994; Scarpulla, 1996) identified two transcription factors, which were termed nuclear respiratory factor 1 (NRF1) and nuclear respiratory factor 2 (NRF2). They appear to be key transcriptional activators of nuclear genes which encode a wide range of mitochondrial enzymes. Nuclear respiratory factor 2 (NRF2), also referred to as the GA-binding protein (GABP) transcription factor, is a key transcriptional activator of many nuclear genes which encode a wide range of mitochondrial enzymes. NRF2 is a complex protein, which consists of an alpha and beta subunit that are encoded by separate genes e.g *GABPA* and *GABPB1*, respectively. The NRF2 belongs to the Cap-N-Collar transcription factor family which recognizes the antioxidant response element (ARE) in the promoter of several target genes (Yu and Kensler, 2005). Binding sites for NRF2 protein have been identified in several nuclear genes including respiratory genes, genes for heme biosynthesis, mitochondrial protein import, as well as mitochondrial DNA transcription, translation, and replication, implicating the NRF2 in the regulation of mitochondrial biogenesis (Baar, 2004).

Previous studies have suggested that the β1-subunit of the NRF2 is encoded by the *GABPB1* gene, which is located on chromosome 15q21.2. The polymorphisms located in this gene region may be associated with increased maximal oxygen uptake (VO_2max_) in response to physical exercise (Bouchard et al., 2000). He et al. (2007) have suggested that the A/G polymorphism in intron 3 of the GABPB1 gene (rs7181866) is associated with oxygen uptake in response to training among Chinese men. Additionally, Eynon et al. (2009) have revealed that AG genotype of the rs7181866 may induce a greater increase in gene transcription and higher protein mRNA expression, and is more frequent in Israeli endurance athletes, particularly in the elite group. They have also suggested that the G allele is associated with higher values of oxygen uptake in response to endurance training.

The aim of this study was to analyze the possible importance of the A/G polymorphism (rs7181866) in intron 3 of *GABPB1* gene in Polish rowers and sedentary individuals, revealing the possible relationships between genotype and physical performance.

## Material and Methods

### Ethics Committee

The Pomeranian Medical University Ethics Committee approved the study and written informed consent was obtained from each participant.

### Subjects and controls

Fifty-five male Polish rowers were recruited for this study. They were divided into two groups: (1) elite rowers (high elite + elite athletes) and (2) non-elite rowers (sub-elite + average athletes) based on the set of definitions for describing athletic status taken from Druzhevskaya et al. (2008). From the group of 30 elite rowers, none with less than ten years of training experience, each athlete won at least one medal. Control samples were prepared from 130 unrelated sedentary volunteers (male students, aged 19–23). The athletes and controls were all Caucasian to prevent any likely racial gene skew and to overcome potential problems of population stratification.

### Genotyping

Genomic DNA was extracted from the oral epithelial cells using a GenElute Mammalian Genomic DNA Miniprep Kit (Sigma, Germany) according to the manufacturer’s instructions.

Genotyping of the *GABPB1* gene A/G polymorphism (rs7181866) was performed using polymerase chain reaction (PCR). The resulting PCR products were genotyped by restriction fragment length polymorphism (RFLP) as recommended by He et al. (2007).

A 483 base pair (bp) fragment of *GABPB1* gene was amplified by PCR using forward primer 5′-AGTTTAGTGTCTCCCAGTGT-3′ and reverse primer 5′-CTTAGTTTTCTTGTATCCGT-3′. The PCR mixture (total volume 20 μl) contained 0.2 mmol/L of each primer (Genomed, Poland), 200 mM each dNTP (Novazym, Poland), 0.5 U Taq polymerase (Novazym, Poland), and 100 ng of genomic DNA. The thermal-time PCR was as follows: initial denaturation at 94°C for 5 min, 35 cycles of denaturation at 94°C for 1 min, primer annealing at 50°C for 1 min, chain extension at 72°C for 1 min and final extension at 72°C for 10 min. The amplified fragments were digested by *RsaI* enzyme (Fermentas, Lithuania) in conditions recommended by the supplier to 483 bp in the presence of the G allele, and 248 bp and 199 bp in the presence of the A allele. The digested products were visualized by 3% agarose gel electrophoresis.

### Statistical Analysis

The STATISTICA statistical package, version 7.0, was used to perform all statistical analysis.

Genotype distribution and allele frequencies between the groups of athletes and controls were compared and significance was assessed by a χ^2^ test. If observed or expected values included a cell with a value < 5, we used Fisher’s exact test to compare alleles and genotype frequencies. The level of statistical significance was set at p < 0.05.

## Results

*GABPB1* genotype distributions amongst subjects and controls were in Hardy-Weinberg equilibrium, making selection bias less likely. The distributions of the *GABPB1* genotypes and alleles are given in Table 1.

Statistically significant differences in genotype distribution were observed when only the whole group of rowers (89.09AA; 10.91% AG, 0% GG; p=0.012) were compared with controls. The genotype distribution amongst elite rowers (90.00% AA, 10.00% AG, 0% GG; p=0.080) and non-elite rowers (88.00% AA, 12.00% AG, 0% GG; p=0.053) were not significantly different from those of sedentary controls. The differences between elite rowers and non-elite rowers were also insignificant (p=0.812).

A significant increase of the G allele frequency was observed in the whole group of rowers compared with controls (5.5% vs. 1.17%, p=0.014). The differences of G allele frequency among elite rowers (5.00%, p=0.082) and non-elite rowers (6.00%, P=0.054) were not statistically significant compared to controls.

## Discussion

Data concerning the *GABPB1* gene in a sport are still limited. Moreover, the hypotheses referencing the role of this gene in physical performance, particularly in endurance sports has not been clearly proven. The present study is the first report regarding the A/G polymorphism (rs7181866) in intron 3 of the *GABPB1* gene in Polish rowers.

In this study, we investigated the association between the *GABPB1* A/G polymorphism and endurance performance. The *GABPB1* gene was taken into consideration as a genetic marker of endurance because of its proposed role in acting on the majority of nuclear genes which encode a wide range of mitochondrial enzymes. It has been suggested that the NRF2 protein improves respiratory capacity and increases the rate of ATP production during exercise (Kelly and Scarpulla, 2004; Osburn and Kensler, 2008), because of its significant role in inducing mitochondrial biogenesis (Kelly and Scarpulla, 2004; Scarpulla, 2002). NRF2 has been shown to play a key role in inducing anti-oxidative enzymes and genes against oxidative stress (Kobayashi and Yamamoto, 2005; Numazawa and Yoshida, 2004). Additionally, increased protein expression has been revealed to decrease pro-inflammatory mediator expression in mice (Khor et al., 2006). An increased expression of the NRF2 protein is probably favorable for endurance performance, as the magnitude of the oxidative stress and the inflammatory response caused by acute exercises which puts a considerable strain and decreases the recovery ability (Nikolaidis et al., 2008; Powers and Jackson, 2008; Suzuki et al., 2004).

The *GABPB1* A/G polymorphism is located in a non-coding region of the gene and it is very interesting, yet unclear how this polymorphism affects gene regulation and protein expression. The mutations in an intron region, such as the *GABPB1* A/G polymorphism, may modify the splicing of mRNA, which may lead to differences in expression and hence an effect on biogenesis of mitochondria (He et al., 2007).

In the present study, we have revealed statistically significant differences in the *GABPB1* genotype distribution and the A/G allele frequency between the group of the endurance athletes and the sedentary controls. 10.91% of the endurance athletes exhibited the AG genotype compared with only 2.31% in the control group. The *GABPB1* AG genotype and the G allele are overrepresented among the Polish rowers, suggesting that this polymorphism may affect endurance performance.

Our data are supported by other authors who have reported associations between the G allele and increased values of oxygen uptake in response to training (He et al., 2007). Additionally, Eynon et al. (2009) revealed that the *GABPB1* (referred to by authors as the *NRF2* gene) AG genotype and the G allele may induce a higher increase in gene transcription and protein mRNA expression, and are more frequent in Israeli endurance athletes, particularly in the elite group. They suggested that this genotype, together with the peroxisome proliferator-activated receptor gamma co-activator 1-alpha gene (*PPARGC1A*) Gly/Gly + Gly/Ser genotypes, may be the “optimal genotype” for endurance athletes. Eynon et al. (2010) have also established that other polymorphisms in this gene as A/C (rs12594956) and C/T (rs8031031), separately and in combination, play an important role in athletic ability. They have suggested that the *GABPB1* AA and CT genotypes may stimulate a higher expression of the NRF2 protein, and thus, possessing these genotypes may confer an advantage to endurance athletes. These conclusions seem to be supported by He et al. (2007), who have examined the *GABPB1* polymorphisms: rs12594956, rs8031031, rs7181866 and demonstrated that Chinese men carrying the ATG haplotype had higher training response in VO_2max_ at RE than non-carriers, suggesting that the *GABPB1* polymorphisms may explain some of the individual endurance abilities.

In our study, we did not identify GG genotypes in either the rowers or the control group. This finding seems to be supported by He et al. (2007), Eynon et al. (2009), and data from the NCBI SNP database (http://www.ncbi.nlm.nih.gov/projects/SNP) regarding Chinese, Israeli, and European populations.

The present study was not without limitations. The investigated group was not large enough, owing to limitations imposed by the small number of rowers who are available in Poland and agreed to participate in our research. Unfortunately, the relatively small size of the group in this study may cause ambiguity of the obtained results (relatively low statistical test power). In addition, athletic performance is a polygenic trait; over 20 polymorphisms have been associated with elite endurance performance (Eynon et al., 2011). The genetic marker analysed independently is likely to make a limited contribution to an “elite phenotype”: it seems more likely that such status depends on the simultaneous presence of multiple such variants (Ahmetov et al., 2009). The other polymorphisms in this gene as A/C (rs12594956) and C/T (rs8031031) and the other genetic markers as *PPARGC1A* may also play important roles in endurance ability and should be taken under consideration in further studies.

In conclusion, our results indicate a higher frequency of the *GABPB1* AG genotype in the group of rowers than the control group. This data may suggest that the G allele is associated with higher endurance capability, and therefore it may be taken into consideration for inclusion in the group of performance enhancing polymorphisms as a factor beneficial to endurance performance. However, these conclusions should be supported with more experimental studies on other *GABPB1* polymorphisms and other such genes which may describe a common phenotype among elite cndurance athletes.

## Figures and Tables

**Table 1:** The GABPB1 A/G genotype and allele frequencies in rowers and controls

Group	N	*GABPB1* genotype			P^*^	*GABPB1* allele		P
		AA	AG	GG		A	G	
All rowers	55	49	6	0	0.012	104	6	0.014
Elite rowers	30	27	3	0	0.080	57	3	0.082
Non-elite rowers	25	22	3	0	0.053	47	3	0.054
Controls	130	127	3	0	-	257	3	-

* AA vs. AG+GG

Genotype distribution: all rowers vs. controls χ^2^ =4.45, df = 1; elite rowers vs. controls χ^2^ =2.148962, df = 1; non-elite rowers vs. controls χ^2^ =3.01, df = 1 Allele frequency: all rowers vs. controls χ^2^ =4.34, df = 1; elite rowers vs. controls χ^2^ = 2.107, df =1; non-elite rowers vs. controls χ^2^ =2.95, df = 1,
